# Randomised Controlled Trial of Fish Oil Supplementation on Responsiveness to Resistance Exercise Training in Sarcopenic Older Women

**DOI:** 10.3390/nu14142844

**Published:** 2022-07-11

**Authors:** Natália Maira da Cruz Alves, Karina Pfrimer, Priscila Carvalho Santos, Ellen Cristini de Freitas, Thiago Neves, Rodrigo Antônio Pessini, Márcia Varella Morandi Junqueira-Franco, Marcello H. Nogueira-Barbosa, Carolyn Anne Greig, Eduardo Ferriolli

**Affiliations:** 1 Department of Internal Medicine, Ribeirão Preto Medical School, University of São Paulo (USP), Ribeirão Preto 14049-900, SP, Brazil; kpfrimer@fmrp.usp.br (K.P.); thiagonevesufmt@gmail.com (T.N.); mvmjf@hotmail.com (M.V.M.J.-F.); eferriol@fmrp.usp.br (E.F.); 2 Department of Nutrition, University of Ribeirão Preto, Avenue Costábile Romano 2201, Ribeirão Preto 14049-900, SP, Brazil; 3 Department of Food and Nutrition, School of Pharmaceutical Sciences of Araraquara State, University of Sao Paulo, Araraquara 14801-902, SP, Brazil; priscilacarvalho.nutri@yahoo.com.br (P.C.S.); ecfreitas@yahoo.com (E.C.d.F.); 4 School of Physical Education and Sport of Ribeirao Preto, University of Sao Paulo, Ribeirao Preto 14049-900, SP, Brazil; 5 Department of Health Sciences, Ribeirao Preto Medical School, University of Sao Paulo, Ribeirao Preto 14049-900, SP, Brazil; 6 Department of Medical Images, Hematology and Clinical Oncology, Ribeirão Preto Medical School, University of São Paulo (USP), Ribeirão Preto 14049-900, SP, Brazil; rodpessini@gmail.com (R.A.P.); marcello@fmrp.usp.br (M.H.N.-B.); 7 School of Sport, Exercise and Rehabilitation Sciences and MRC-Versus Arthritis Centre for Musculoskeletal Ageing Research, University of Birmingham, Birmingham B15 2TT, UK; c.a.greig@bham.ac.uk

**Keywords:** sarcopenia, fish oil, muscle strength, aging

## Abstract

This study aims to investigate the effects of fish oil supplementation on the muscle adaptive response to resistance exercise training, physical performance and serum levels of inflammatory cytokines in sarcopenic older women. A randomised, double-blind, placebo-controlled trial is performed with thirty-four sarcopenic women (2010 European Consensus of Sarcopenia), aged ≥ 65 years. The participants are allocated into the following two groups: Exercise and Fish Oil (EFO) and Exercise and Placebo (EP). Both groups undertook a resistance exercise programme over 14 weeks. All participants are instructed to ingest 4 g/day of food supplements; the EP group received sunflower oil capsules, and the EFO group, fish oil capsules. The cross-sectional area (CSA) of the quadriceps muscle is calculated using magnetic resonance imaging (MRI). The strength of the lower limbs is measured using isokinetic dynamometry. Both groups show improvements in CSA and strength after the intervention. Changes in EFO are significantly greater compared with EP for muscle strength (peak torque, 19.46 Nm and 5.74 Nm, respectively, *p* < 0.001). CSA increased after the intervention in both groups (EFO; 6.11% and EP; 2.91%), although there is no significant difference between the groups (*p* = 0.23). There are no significant intra-group, inter-group or time differences in any of the cytokines measured. The use of fish oil supplementation potentiates the neuromuscular response to the anabolic stimulus from training, increasing muscle strength and physical performance in sarcopenic older women.

## 1. Introduction

Sarcopenia, defined as the age-related reduction of muscle mass and function [1,2,3], is associated with negative health outcomes and considerable social and economic impacts [4].

The decline in physical activity, which accompanies advancing age, is considered one of the main factors influencing the development of sarcopenia [5,6]. A sedentary lifestyle is one of the main contributors to this condition, and higher levels of physical activity may attenuate its progression [7,8].

Muscle loss has been reported to vary between the sexes [9]. Women have physiologically lower muscle mass, strength and physical function for a given age compared with men throughout their lifetime; however, the respective rates of decline of these parameters do not differ [10]. However, Lynch et al. report that sex differences may be dependent upon anatomical location (upper versus lower limb) and the method of measurement of muscle function [11].

It has been well established in the literature that progressive resistance exercise training (RET) improves muscle strength, mass and functional performance in older people [12,13,14,15], as it promotes morphological and functional adaptations in skeletal muscle [16]. It is therefore the key modality for the management of sarcopenia [17]. 

Omega-3 polyunsaturated fatty acid supplementation has been shown to decrease blood pressure [18]. In addition, previous research has shown that fish oil supplementation combined with RET may potentiate anabolic responsiveness [19,20,21]. This could be due to an increase in the availability of amino acids, increasing myofibrillar protein synthesis; an increase in nerve conduction speed [12,20] and the improvement in the modulation of ionic calcium channels of the sarcolemma [12,22,23,24]. Again, there may be sex differences in the response to supplementation, with one study showing increases in muscle function and quality in older women but not in older men [21]. 

Most of the studies involving fish oil supplementation and RET have been performed in younger adults and older people in whom sarcopenia was not evaluated. Considering the lack of studies including sarcopenic sedentary older adults and women in particular, this study aimed to investigate the effect of a 14-week period of omega 3 polyunsaturated fatty acid supplementation on the responsiveness to RET in sedentary older sarcopenic women.

## 2. Materials and Methods

### 2.1. Participants

This study was approved by the Human Research Ethics Committee of the Clinics Hospital of the Ribeirao Preto Medical School, University of Sao Paulo (5607/2016) and registered at Clinical Trials.gov (NCT3462771).

The participants were recruited at the Geriatric Medicine Outpatient Clinic of the Clinics Hospital of the Ribeirao Preto Medical School, Family Health Centres and in the community of the city of Ribeirao Preto. 

Inclusion criteria were female, aged 65 years or older, sarcopenic according to the EWGSOP (2010) criteria [25], sedentary (defined as performing moderate to intense physical activity for less than 150 min/week during the last three months) and having BMI below 30 kg·m^−2^. Exclusion criteria included use of any fish oil supplements, any acute or chronic disease or condition that would adversely affect participant safety and/or ability to continue with the RET programme, as identified by the application of the Physical Activity Readiness Questionnaire (PAR-Q).

### 2.2. Randomisation and Blinding

Participants were randomly allocated (1:1) to Group Exercise and Fish Oil (EFO) n = 17 and Group Exercise and Placebo (EP), *n* = 17, using the Research Randomizer software (version 4.0) [26]. Identical bottles containing capsules of fish oil (Eicosapentaenoic acid (EPA); 440 mg and Docosahexaenoic acid (DHA); 220 mg) or sunflower oil (as placebo) were provided. Participants, evaluators and researchers were blinded to the treatment, to maintain the double-blind study design.

### 2.3. Screening for Sarcopenia

The EWGSOP (2010) [27] criteria were followed for the diagnosis of sarcopenia. Muscle mass was estimated using bioelectrical impedance analysis, muscle strength using handgrip dynamometry and physical performance by the measurement of walking speed, as described below.

#### 2.3.1. Skeletal Muscle Mass Index

The assessment of body composition was performed, as part of the screen for sarcopenia status, using a portable electric bioimpedance device, tetrapolar, ImpediMed Pty Ltd., model ImpDF 50 (Pinkenba, Queensland, Australia). The procedures indicated by the equipment manual for the examination were followed. The resistance values obtained were used to calculate skeletal muscle mass (SMM), using the formula, SMM = [(h^2^/R × 0.401) + (sex × 3.825) + (age × −0.071)] + 5.102, with height (h) in centimeters, “R” being the resistance value in ohm; for sex, woman = 0 and man = 1, age in years. The skeletal muscle mass index (SMI) was calculated by dividing SMM by the square of the height [23]. The cut-off value of SMI for the diagnosis of sarcopenia was ≤6.75 kg·m^−2^.

#### 2.3.2. Handgrip Strength (HGS)

A portable hydraulic dynamometer (Saehan Corporation, Masan Free Trade Zone, Masan, Korea) was used for the HGS test. The test was applied according to the recommendations of the American Society of Hand Therapists (ASHT) [28]. The participant was instructed to remain sitting comfortably with an arm adducted parallel to the trunk, the elbow flexed at 90°, the forearm in a neutral position and the wrist in 0° to 30° extension. The participant was instructed and encouraged to pull the loop of the dynamometer, maintaining for six seconds and then relax. Three measurements were performed at one-minute intervals, with the participant alternating between the dominant and non-dominant side. The average of three measurements was used in subsequent analysis.

#### 2.3.3. Gait Speed

Gait speed was measured over a 4 m course. Participants were instructed to stand with both feet touching the starting line and to begin walking at their usual pace after a verbal command. The average of two walks was used to compute the measure of gait speed [29].

### 2.4. Measurement of Functional Capacity

#### 2.4.1. Six-Minute Walking Test

The six-minute walking test (6mWT) was applied before and after the intervention, in order to evaluate the physical capacity of the volunteers [30,31]. The test was performed according to the American Thoracic Society (ATS) [32] 2002 guideline. The test was performed in a hospital corridor. A 30 m distance, measured with an inextensible metric tape, was marked with colour tape every 3 m. Each participant was instructed to walk along the marked distance, as fast as she could, during the 6 min. The distance walked was recorded in meters.

#### 2.4.2. Lower Limb Muscle Strength (Peak Torque) and Power

The peak torque and power of the lower limbs were measured before and after the intervention using dynamometry (Biodex 4 Pro, Biodex, Shirley, New York, NY, USA) at angular speeds of 60º/s and 180º/s. The participants were encouraged to perform five maximum extensions for each lower limb, with a resting time of at least 30 s between each extension. The highest value was recorded and used in subsequent analysis.

#### 2.4.3. Quadriceps Cross-Sectional Area (CSA) Using MRI

For the acquisition of magnetic resonance images, the Philips Achieva Intera—1.5 T equipment (Caserta, Italy) was used, allocated at HCMRP –USP. During the measurements, the participants were instructed to remain lying in the supine position with their arms relaxed at their sides and immobile.

In order to facilitate the location of the quadriceps muscle and the axial cut of the images for analysis, an Advil™ (Pfizer, Itapevi, São Paulo) capsule was fixed with tape as a marker at a midpoint of the right thigh located between the upper edge of the patella and the anterosuperior iliac spine [33].

The protocol adopted for the sequence of images was the same used in the service routine of the Radiology Department of the Clinics Hospital, and consisted of a T1 sequence with turbo spin-echo with axial cut of TR = 500 ms; TE = 15 ms; cutting thickness of 8 mm; 240 × 240 matrix; FOV AP 240, RL 240, FH 272 mm, image size (X, Y, Z) 240 × 240 × 34 and voxel size (X, Y, Z) of 1 × 1 × 8 mm. All segmentations were performed by one previously trained examiner (NMCA), bordering the internal edge of the muscular fascia from the axial T1-weighted image. 

For the analysis and segmentation of the images, the 3D Slicer software (version 4.10.2, Tres Cantos, Spain)^®^ was used, an 8 mm segment was analyzed, using the image with Advil capsule as reference. Thus, CSA was measured by the ratio between the quads segment volume and its thickness.

#### 2.4.4. Muscle Quality

Lower limb muscle quality was calculated as the ratio of the isokinetic strength of the dominant limb (peak torque Nm) and cross-sectional area (cm^2^) of the same limb [34]. 

### 2.5. Biochemical Evaluation

#### 2.5.1. Inflammatory Cytokines

In order to investigate the presence of inflammation, plasma concentrations of IL1, IL6, IL8, IL10 and TNF-α were measured. The analyses were performed in duplicate, using the multiplex assay panel (HCYTOMAG-60K, Miliplex^®^, kit Merck Milipore, Darmstadt, Germany), using the equipment Luminex^®^ TM Technology (Magpix, Austin, TX, USA). The assays were performed according to the manufacturer’s instructions. Cytokine concentration was calculated using the Miliplex Analyst software^®^ (version 5.1, Darmstadt, Germany).

#### 2.5.2. Plasma and Capsules Fatty Acid Concentration

The analysis of plasma samples and the oil in the capsules was performed in duplicate. Total lipids were extracted using the Folch method (1957), and lipids extracted by direct transesterification, as described by Lepage and Roy (1984). The analyses were performed in the mass spectrometry laboratory at FMRP-USP. After extraction, total fatty acids were determined by gas chromatography (SHIMADZU, GC-2010). 

The analysis of the capsules showed the presence of the following fatty acids: myristic (14: 0) 7.61%, palmitic (16: 0) 21.36%, palmitoleic (16: 1n7) 10.77%, steric (18: 0) 4.20%, oleic (18: 1n29) 13.33%, linoleic (18: 2n26) 0.70%, α-linolenic (18: 3n23) 0,83%, arachidonic (20: 4n26) 1.44%, EPA (22: 5n23) 21.31% and DHA (22: 6n23) 12.75%. The following fatty acids were present in the sunflower oil capsule: palmitic (16: 0) 4.51%, steric (18: 0) 3.05%, oleic (18: 1n29) 44, 84% and linoleic (18: 2n26) 47.58%.

### 2.6. Intervention Protocol

All participants were instructed to ingest two capsules of the supplement offered at lunch and dinner (4 g·day^−1^). This was based on the fact that bioavailability of fish oil is optimal when taken with meals [35]. The daily dose of fish oil in the present study was selected on the basis of the previously reported positive effects on muscle mass in healthy older people, thus being physiologically relevant in humans [36]. All capsules were provided by Sorocaps Pharmaceutical Industry Ltd. (Sorocaba, Brazil). Adherence was assessed indirectly, by interview and weekly reinforcement of the importance of the correct use of the supplement during the training sessions and by controlling the number of capsules in each bottle (bottles returned by the participants every 12 days), and directly, by measuring the plasma concentration of fatty acids EPA and DHA in the pre- and post-intervention period. 

The one-repetition maximum test (1RM) [37] was applied for the determination of the training intensity. During the first two weeks, the exercises were conducted at 50% of 1RM, aiming to attain neuromuscular adaptation and attenuation of the risk of muscle soreness and injuries. From the third week, intensity was increased to 70% of 1RM and, from the seventh week on, to 80% of 1RM [12]. Training sessions were performed under the supervision of a member of the study team (physiotherapist, NMCA) and one qualified instructor (physical educator, TN). Temperature at the gym was controlled at 21 °C. 

Supervised training sessions were held three times per week, in the gymnasium of the School of Physical Education and Sports of the University of Sao Paulo in Ribeirao Preto. The participants were instructed and encouraged to perform three series of 12 repetitions for each exercise, with one minute of rest between each series. Both the eccentric and concentric phases were included, over the full range of movement. At the beginning of each session an aerobic warm-up session was performed for 10 min.

The following exercises were included: Leg extension, 45° Leg Press, Horizontal Leg Press, bilateral Knee Flexion with a shin weight, Hip Abduction and Hip Adduction. 

### 2.7. Statistical Analysis

The calculation of sample size was based on peak torque with an effect size of 0.7. Pre- and post-intervention values were extracted from Rodacki et al. [12] The minimum sample size to detect a significant difference in peak torque with 90% power required *n* = 24 (12 in each group), and considering an estimated attrition of up to ten participants, we defined a final sample size of 34 older women. 

The R Core Team software (Statistical Computing, Vienna, Austria) was used to analyze descriptive data, body composition and inflammatory cytokines. A linear regression mixed-effects model was used to compare within- and between-group differences over time. Mean estimated differences with their respective 95% confidence intervals were reported. The SAS Statistical software (version 9.3; SAS Institute Inc., Cary, NC, USA) was used for this analysis. 

GLM repeated ANOVA analyses were employed with group and time as factors, to verify within/between-group differences and interactions in functional performance parameters, using the SPSS software (version 25.0). 

To analyze the fatty acid profile pre- and post-intervention, the paired Student’s *t*-test was applied, using the SPSS software (version 25.0). For all analyses, the 5% level of significance was adopted.

## 3. Results

The daily dose of fish oil in this study (4 g·day^−1^) was not associated with any adverse effects during the 98 days of supplementation.

**Figure 1:**
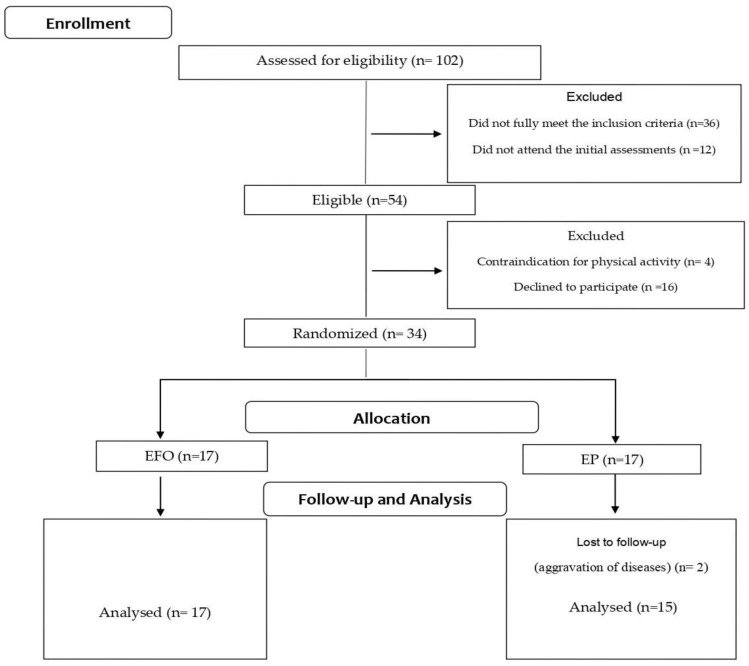
The most prevalent comorbidities were diabetes mellitus and high blood pressure.

One hundred and two older women were initially identified as potentially eligible for participation (Figure 1).

Forty-eight women who did not fully meet the inclusion criteria or who did not attend the initial interview were excluded. Fifty-four women fulfilled the inclusion criteria and were invited to participate in the study. Of those, 16 refused participation due to the time commitment needed and four women were excluded due to the presence of contraindications for RET.

Thus, thirty-four older women were included and underwent a clinical assessment and blood tests to verify their health status. 

After the first week of intervention, two participants in the placebo group withdrew from the study due to aggravation of existing health conditions.

The general and sociodemographic characteristics of the volunteers included are shown in Table 1.

The body composition of both groups of participants, in the pre- and post-intervention periods, is presented in Table 2. There was a significant increase in BMI and SMI in both groups post-intervention. There was a significant increase in body weight only in the fish oil group post-intervention.

Table 3 shows the data for the physical performance and muscle parameters. There were significant differences between pre- and post-intervention in handgrip strength (*p* < 0.001), 6mWT (*p* = 0.008) and muscle quality (*p* = 0.002). There was a significantly greater increase in the distance walked in the 6mWT in the EFO group compared with the EP group (*p* = 0.009) and a greater increment in muscle quality in the EFO group (12.31%) compared with the EP group (4.22%) (*p* = 0.004). Peak torque increased after the intervention in both groups (*p* < 0.001) and the increment was significantly higher in the EFO group (59.75 Nm to 79.21 Nm; 32.5%) compared with the EP group (63.92 Nm to 69.66 Nm; 8.98%), *p* = 0.003.

Table 4 shows the plasma concentration of inflammatory cytokines before and after the intervention. There were no significant intra-group, inter-group or time differences in any of the cytokines measured.

**Figure 2:**
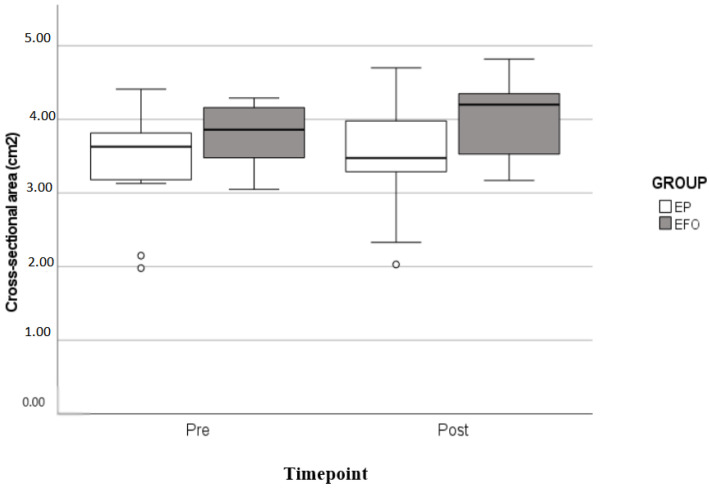
Cross-sectional area of the quadriceps muscle of both groups in the pre- and post-intervention period. GLM repeated measures ANOVA.

The quadriceps cross-sectional area (Figure 2) increased after the intervention in both groups (*p* = 0.006). In the EFO group, the increment was 6.11% (from 3.76 cm^2^ to 3.99 cm^2^) and in the EP group, 2.91% (from 3.44 cm^2^ to 3.54 cm^2^); however, there was no statistically significant difference between groups (*p* = 0.23).

Regarding the fatty acid profile, there was a significant increase in the concentrations, in the post intervention period, of EPA (0.32% ± 0.73% and 1.27% ± 0.88%, respectively, *p* < 0.001) and DHA (0.70% ± 0.84% and 1.95%± 0.60%, respectively, *p* < 0.001) in the EFO group. In the EP group, there was no significant change in the fatty acid profile between periods (0.20% ± 0.38% pre intervention to 0.22% ± 0.43% post intervention, *p* = 0.92) for EPA and (0.71% ± 0.67% pre intervention to 0.68% ± 0.79% post intervention, *p* = 0.92) for DHA.

## 4. Discussion

This study aimed to investigate the effect of fish oil supplementation on the adaptive muscle response to resistance exercise training in sarcopenic older women. Although other studies have investigated the effect of fish oil supplementation associated with resistance training on muscle responsiveness, they were performed in older people whose sarcopenia status was unknown [12,36]. Moreover, there have been no studies to date on a Brazilian population.

The main result of our study was that although participants from both groups showed an increase in muscle strength and CSA after physical training, the group supplemented with fish oil had a higher increment (23.6% higher in peak torque and 3.2% in CSA).

Several mechanisms are involved in the improvement of muscle strength, and the initial gains can be attributed to the neural adjustment that occurs during the first weeks of training, which is followed by an increase in the cross-sectional area of the muscle [12]. Although both strength and CSA showed a slightly greater increase in the group supplemented with fish oil compared with the placebo, the mechanisms of the effect of fish oil supplementation on muscle are still not completely understood. According to recent investigations [37,38], there are the following three possibilities to explain these benefits: anti-inflammatory effects, reduction of insulin resistance and increased activation of the mTOR pathway. 

The anti-inflammatory effects of fish-oil supplementation have already been well described in older adults. A recent randomised controlled study including *n* = 35 older people investigated the effect of supplementation with EPA and DHA on inflammatory cytokine plasma levels. Supplementation for at least four weeks was associated with a significant reduction in IL-6, IL-1β and TNFα [39].

In contrast, in the present study, supplementation with omega-3 fatty acids was not associated with significant changes in the plasma concentration of inflammatory cytokines. These results are consistent with previously published experimental studies where the anabolic effects of omega-3 fatty acids were independent of any significant influence on inflammation [16,19,40]. 

Previous studies suggest that there is an important decrease in insulin resistance in response to omega-3 supplementation. As insulin signalling has an important role in the activation of mTOR, it is possible that supplementation with omega-3 PUFA may mitigate metabolic resistance and stimulate protein synthesis in older people [38,41]. However, further studies are needed to clarify how omega-3 supplementation affects insulin sensitivity and this was not an aim of this study. 

Evidence to date suggests that the activation of mTOR is the most probable explanation for the action of fish oil on muscle mass [19]. A recent study including sixteen healthy older adults showed higher concentrations of mTORSer2448 and p70s6kThr389 after supplementation with omega-3 fatty acids (4 g·day^−1^) for eight weeks. The authors proposed that the effect was at least partially mediated via the enhanced activation of mTOR-p70s6k (target of rapamycin in mammals/kinase ribosomal protein S6), a signalling pathway that is known to influence skeletal muscle mass, particularly under mechanical stimulation conditions [19].

Another important finding of this study regards muscle strength. As a result of the resistance exercise training, both groups showed a significant increase in both lower limb strength (peak torque) and handgrip strength. However, the group supplemented with fish oil showed a greater increase in peak torque when compared with the placebo group. 

The changes in peak torque in our study were lower than those reported in other studies involving resistance training and fish oil supplementation. Rodacki et al. [12], for example, showed an increase of 50% in knee extensor peak torque in older women supplemented with fish oil when compared with a placebo. The smaller increase found in our study may have been due to differences in the studied population; sarcopenic older women may have had a higher resistance to anabolic stimuli.

Both groups showed statistically significant increases in handgrip strength. Barbosa et al. [42] showed that a 10-week resistance exercise training programme resulted in an increase in the handgrip strength of older women. Although the present study included exercises for the lower limbs only, handgrip strength reflects global strength and has been shown to be improved as a consequence of resistance training of the lower limbs [43].

There was no difference between the two groups in physical capacity as evaluated by the 6-min walking test at baseline. After the intervention, the supplemented group showed a significantly greater increase in the distance walked (*p* = 0.009). These results agree with those of Rodacki et al. [12], who also described a significant increase in groups supplemented with fish oil compared with placebo.

Muscle quality has been suggested to be a better muscle health biomarker than muscle mass because it represents an estimation of the neural and morphologic factors influencing strength [43,44]. In the present study, muscle quality improved significantly after the intervention in both groups, but the older women supplemented with fish oil showed a larger increment in muscle quality (EFO group, 12.31% versus 4.22% in the EP group; *p* = 0.004). 

Muscle power did not increase significantly in both groups after the intervention. Although a number of studies have shown that resistance training is associated with an increase in muscle power [45,46,47,48], this effect depends specifically on the training pattern. i.e., it needs to incorporate high-speed contractions [42], which were not included in our training regimen. 

Gait speed also did not increase after 14 weeks of training in both groups, although the difference was borderline significant for both (*p* = 0.059).

However, according to a previous systematic review [49], changes in gait speed of 0.10–0.20 ms^−1^ may be considered clinically relevant in some groups of patients, including older people. In the current study, 47% (*n* = 8) of the older women in the EFO group and 40% (*n* = 6) of the EP group presented with a post-intervention increase in gait speed, which could be considered clinically significant.

Previous studies have shown positive effects of resistance training on gait speed and stability [50,51,52,53]. However, consistent with our findings, Sullivan et al. [54] demonstrated that resistance training does not significantly increase gait speed, indicating the potential influence of other important variables involved in walking performance, such as those associated with sensory and vestibular function. 

Studies involving resistance training in older adults [12,45] included 12-16 weeks of training. However, according to Lixandrao et al. [55], the studies on the RET time course for the induction of hypertrophy were mostly based on younger populations. According to the authors, 18 RET sessions, that is, nine weeks, are enough to induce muscle hypertrophy in older people. 

The present study has some limitations. These include the relatively small sample size, which was based on estimated differences in peak torque from a previous study [12]. In addition, restricting the participants to older women limited the generalizability of the results. Further studies involving a larger number of both older women and men are needed.

## 5. Conclusions

The use of fish oil supplementation concomitantly with strength training potentiates neuromuscular response to the anabolic stimulus from training, increasing muscle strength and physical performance in sarcopenic elderly women.

Omega-3 fatty acid supplementation may provide a safe, simple solution and low-cost intervention to counteract muscle loss and its complications in conditions associated with sarcopenia. Furthermore, resistance training remains the key element for increasing strength and muscle mass in the sarcopenic elderly.

## Figures and Tables

**Table 1:** General and sociodemographic characteristics of the included volunteers (*n* = 32).

Age (Years), Mean (SD)	
Age (Years), Mean (SD)	
EP	71.4 (6.21)
EFO	70.6 (3.94)
Ethnicity, n (%)	
White	22 (68.7%)
Black	2 (6.25%)
Eastern	8 (25.0%)
Marital status, n (%)	
Married	11 (34.4%)
Widow	13 (40.6%)
Divorced	8 (25.0%)
Educational level (years), n (%)	
1–4 years	17 (53.1%)
>4 years	15 (46.9%)
Number of medications per day, mean(SD)	
EP	2.7 (3.9)
EFO	2.3 (2.4)
Comorbidities, n (%)	
High blood pressure	
EP	13 (86.7%)
EFO	14 (82.6%)
Diabetes mellitus	
EP	5 (33.3%)
EFO	9 (52.9%)

EP: Exercise and Placebo Group; EFO: Exercise and Fish Oil Group.

**Table 2:** Body composition and height of the participants in the pre- and post-intervention periods (*n* = 32).

	Group	Pre-Intervention Mean (SD)	Post-Intervention Mean (SD)	Estimated Difference	95% Confidence Interval	*p* Value
Weight (kg)	EP	57.9 (11.8)	59.3 (12.5)	−1.3	−3.03; 0.42	0.13
	EFO	62.5 (8.5)	64.8 (10.7)	−2.4	−3.99; −0.75	0.01 ^†^
Height (m)	EP	1.52 (0.09)	1.51 (0.08)	0.02	0.01; 0.03	0.00 ^†^
	EFO	1.56 (0.06)	1.55 (0.05)	0.02	0.01; 0.03	0.00 ^†^
BMI (kg·m^−2^)	EP	24.73 (3.48)	25.95 (4.21)	−1.22	−1.97; −0.48	0.00 ^†^
	EFO	25.58 (3.07)	27.11(3.95)	−1.53	−2.23; −0.84	0.00 ^†^
SMI (Kg/m^−2^)	EP	6.16(0.39)	7.12 (0.92)	−0.95	−1.66; −0.25	0.01 ^†^
	EFO	6.17(0.48)	8.38(1.52)	−2.21	−2.86; −1.56	0.00 ^†^

Linear regression mixed effects model. **^†^** Significance at *p* < 0.05 level, BMI: body mass index; SMI: skeletal muscle index.

**Table 3:** Comparison of changes in physical performance and functional parameters within and between groups (*n* = 32).

	Group	Pre-Intervention Mean (SD)	Post-Intervention Mean (SD)	*p*-Value Time	*p*-Value Time * Group
HS (kgf)	EP	18.58 (4.28)	21.72 (3.00)	0.000 ^†^	0.843
	EFO	20.75 (5.47)	24.14 (4.14)		
GS (m/s)	EP	1.22 (0.48)	1.36 (0.41)	0.059	0.655
	EFO	1.11 (0.38)	1.19 (0.35)		
6mWT (m)	EP	376.60 (107.2)	377.20 (105.5)	0.008 ^†^	0.009 ^†^
	EFO	378.20 (60.64)	443.10 (101.6)		
MQ (Nm/cm^2^)	EP	18.54 (4.44)	19. 32 (5.05)	0.002 ^†^	0.004 ^†^
	EFO	17.77 (4.72)	19.92 (4.38)		
Power (watts)	EP	31.7 (10.5)	33.0 (14.2)	0.641	0.635
	EFO	31.5 (9.07)	29.9 (12.6)		
Peak Torque (Nm)	EP	63.92 (19.48)	69.66 (21.84)	0.000 ^†^	0.003 ^†^
	EFO	59.75 (24.59)	79.21 (18.39)		

GLM repeated ANOVA, ^†^ Significance at *p* < 0.05, HS: Handgrip Strength; GS: Gait Speed; 6 mWT: six-minute walking test; MQ: muscle quality. * GLM repeated ANOVA was used to identify differences between factors group and time (pre and posttraining).

**Table 4:** Plasma concentration of inflammatory cytokines before and after the intervention (*n* = 32).

	Group	Pre-Intervention Mean (SD)	Post-Intervention Mean (SD)	Estimated Difference	95% Confidence Interval	*p* Value
IL1_β_ (pg/mL)	EP	0.87 (0.91)	0.73 (0.54)	0.13	−0.22; 0.49	0.45
	EFO	0.68 (0.57)	0.67 (0.28)	0.01	−0.33; 0.35	0.94
IL6 (pg/mL)	EP	2.72 (2.35)	1.93 (1.86)	0.79	−0.93; 2.51	0.36
	EFO	5.55 (7.02)	4.19 (5.68)	1.36	−0.31; 3.02	0.11
IL8 (pg/mL)	EP	4.48 (1.79)	5.82 (5.44)	−1.34	−3.89; 1.21	0.29
	EFO	5.09 (3.59)	5.45 (2.96)	−0.35	−2.82; 2.11	0.77
IL10 (pg/mL)	EP	4.20 (3.95)	10.41 (26.41)	−6.20	−16.14; 3.73	0.21
	EFO	4.48 (3.27)	4.09 (3.39)	0.32	−9.29; 9.95	0.94
TNFα (pg/mL)	EP	13.70 (7.15)	13.39 (5.94)	0.31	−2.97; 3.59	0.85
	EFO	15.63 (6.40)	14.77 (6.14)	0.86	−2.32; 4.04	0.58

Linear regression mixed effects model.

## Data Availability

The data presented in this study are available on request from the corresponding author. The data are not publicly available due to ethical restrictions.
